# The Role of Stem Cells in the Treatment of Cardiovascular Diseases

**DOI:** 10.3390/ijms25073901

**Published:** 2024-03-31

**Authors:** Estera Bakinowska, Kajetan Kiełbowski, Dominika Boboryko, Aleksandra Wiktoria Bratborska, Joanna Olejnik-Wojciechowska, Marcin Rusiński, Andrzej Pawlik

**Affiliations:** 1 Department of Physiology, Pomeranian Medical University, 70-111 Szczecin, Poland; esterabakinowska@gmail.com (E.B.); kajetan.kielbowski@onet.pl (K.K.); dominikaboboryko@gmail.com (D.B.); olejnikjoanna25@gmail.com (J.O.-W.); rusinski.marcin92@gmail.com (M.R.); 2 Department of Internal Medicine, Poznań University of Medical Sciences, 60-780 Poznan, Poland; aleksandrabratborska@gmail.com

**Keywords:** cardiovascular diseases, stem cells, mesenchymal stem cells, induced pluripotent stem cells

## Abstract

Cardiovascular diseases (CVDs) are the leading cause of death and include several vascular and cardiac disorders, such as atherosclerosis, coronary artery disease, cardiomyopathies, and heart failure. Multiple treatment strategies exist for CVDs, but there is a need for regenerative treatment of damaged heart. Stem cells are a broad variety of cells with a great differentiation potential that have regenerative and immunomodulatory properties. Multiple studies have evaluated the efficacy of stem cells in CVDs, such as mesenchymal stem cells and induced pluripotent stem cell-derived cardiomyocytes. These studies have demonstrated that stem cells can improve the left ventricle ejection fraction, reduce fibrosis, and decrease infarct size. Other studies have investigated potential methods to improve the survival, engraftment, and functionality of stem cells in the treatment of CVDs. The aim of the present review is to summarize the current evidence on the role of stem cells in the treatment of CVDs, and how to improve their efficacy.

## 1. Introduction

Stem cells are self-renewing cells that can differentiate into other cell subtypes. They therefore have important potential for regenerative medicine. These cells differ in terms of differentiation potential, and several subtypes can be distinguished. For instance, pluripotent stem cells have the ability to differentiate towards mesoderm, ectoderm, and endoderm. Multipotent cells include mesenchymal stem cells (MSCs), which can be found in various tissues, and can transform into cells from a particular germ layer [1]. The regenerative properties of stem cells have been investigated in the context of numerous conditions, such as neurodegenerative diseases [2], osteoarthritis [3], and periodontitis [4]. Along with their regenerative potential, stem cells have immunomodulatory properties that have beneficial effects in inflammatory conditions. Cardiovascular diseases (CVDs) represent a major cause of death. In 2019, there were approximately 19 million CVD-related deaths [5]. Multiple therapeutics are currently used in clinical practice to reduce the mortality associated with CVDs, but there is a need for new anti-inflammatory and regenerative agents. The aim of this review is to present and discuss the current evidence on the role of stem cells in the treatment of CVDs. We will focus on the use of mesenchymal stem cells (MSCs) and induced pluripotent stem cells (iPSCs). Currently, iPSCs seem to be the most attractive cells in the field of cardiac regeneration. However, numerous studies examined the potential mechanisms to augment the functionality of MSCs; therefore, we will also discuss methods to further increase their beneficial effects.

## 2. Cardiovascular Diseases—Characteristics and Current Treatment Strategies

Cardiovascular diseases represent a broad group of diseases with a significant global burden. In 2019, there were over 500 million patients with CVDs [5]. This term includes a group of heart and vascular disorders, including atherosclerosis, coronary artery disease (CAD), stroke and cerebral hemorrhage, cardiomyopathies, and heart failure (HF). CVDs frequently coexist and their pathogeneses are related. Major behavioral risk factors for CVDs include poor diet, physical inactivity, tobacco use, and alcohol consumption. These factors may cause high blood pressure, hypercholesterolemia, hyperglycemia and obesity, which are associated with increased risk of heart attack, stroke, and heart failure. In this article, we will focus on CAD, cardiomyopathy, and HF. These conditions are associated with heart remodeling and cardiomyocyte death. Regenerative properties of the heart are limited and involve fibrosis of the area affected by cardiac cells necrosis, a process that aims to preserve organ integrity [6]. 

Coronary artery disease is a medical condition associated with atherosclerotic plaque accumulation on the arterial walls. It leads to decreased blood flow through the arteries, heart muscle hypoxia and coronary insufficiency [7]. CAD encompasses a variety of clinical conditions, including asymptomatic atherosclerosis, stable angina, and acute coronary syndrome (unstable angina (UA), non-ST elevation myocardial infarction (NSTEMI), ST elevation myocardial infarction (STEMI)). The symptoms of stable angina include chest pain, especially related with stress or physical exercise, which is radiant to neck, mandible, or arms. The pain subsides at rest or after nitroglycerin consumption in 1–3 min. Importantly, stable angina does not lead to the necrosis of the cardiac cells. The unstable angina is a more severe medical condition. UA is associated with nitroglycerin- resistant chest pain. It does not deescalate at rest and may occur spontaneously. UA is caused by atherosclerotic plaque damage, which leads to reduced flow through coronary artery. UA may progress to NSTEMI and may be a life-threatening condition. NSTEMI and STEMI are associated with heart muscle cells necrosis. STEMI evolution is more rapid, the clot entirely blocks the blood flow and immediately after 15–30 min provide to heart muscle cells necrosis. The area of NSTEMI is generally less expanded, which is linked to developed collateral circulation. The patients with CAD are classified depending on the symptoms of the disease in four-stage scale of Canadian Cardiovascular Society (CCS). The symptoms are generally associated with exercise capacity, where stage I is the gentlest and not- limiting the everyday activity, and stage IV is linked to symptoms occurrence even at rest [8].

Current strategies of CAD treatment are based on endovascular interventions, coronary artery bypass surgery (CABG) [9], pharmacological and non-pharmacological therapy. Invasive treatments are more effective, but conservative treatment methods are also beneficial. They slow down the progression of atherosclerosis, relieve the symptoms, and prevent atherothrombotic events [10]. These methods are based on the combination of anti-ischemic drugs—primarily beta-blockers or calcium-channel inhibitors—and the ad hoc use of nitrates. Agents with antiplatelet properties, such as acetylsalicylic acid or clopidogrel, reduce the risk of major vascular events. Furthermore, hypercholesterolemia treatment with statins and other lipid-lowering agents reduces the accumulation of atherosclerotic plaque [11]. Lifestyle modification is crucial to reduce cardiovascular risk factors, including diet changes, exercise, and smoking cessation.

Cardiomyopathies are relatively rare medical conditions, divided into the main (genetic, mixed, or acquired) and secondary categories, which result in various phenotypes such as dilated, hypertrophic, and restricted patterns [12]. A diagnosis of dilated cardiomyopathy (DCM) is made in the case of systolic dysfunction and left ventricle (LV) dilation in the absence of CAD. Multiple factors may predispose to the occurrence of DCM, including infections, toxins, and inflammatory and metabolic factors [13]. Hypertrophic cardiomyopathy (HCM) is the most common hereditary heart disease, characterized by left ventricular hypertrophy which can lead to heart failure and acute coronary syndromes [14]. HCM primarily may be asymptomatic or cause arrhythmias, fainting or exercise limitation. First-line pharmacotherapy for patients with HCM are negative inotropic drugs, such as β-blockers or non-dihydropyridine calcium-channel blockers [15]. Other treatment methods involve procedures that reduce the thickness of the septum, such as alcohol ablation or myectomy [16]. Occasionally, patients may require dual-chamber electrostimulation, cardioverter–defibrillator implantation, or even heart transplantation. The decision to implant a cardioverter defibrillator is based on HCM Risk-SCD, which estimates the risk of sudden cardiac death at 5 years in patients with HCM [17].

Heart failure is a medical condition characterized by symptoms induced by a structural or functional cardiac defect, confirmed by high natriuretic peptide levels and/or objective evidence of pulmonary or systemic congestion [18,19]. HF is classified as acute or chronic. The most common causes include hypertension, CAD, cardiomyopathies, valve defects and pericardial diseases. In addition, other extracardiac disorders can contribute to the development of HF, such as diabetes, kidneys failure, infections. Chronic HF is defined by the left ventricle ejection fraction (LVEF) and classified into three groups: a reduced ejection fraction (LVEF ≤ 40%); a mildly reduced ejection fraction (LVEF 41–49%); and a preserved ejection fraction (LVEF ≥ 50%) [20]. Chronic HF major symptoms are dyspnea, exercise limitation, leg swellings, enlargement of abdominal circumference, etc. Patients with HF are classified by New York Heart Association (NYHA scale). The scale divides patients into four groups depending on exercise capacity [21].

Pharmacotherapy of heart failure is based on four main groups of medications. The first is renin–angiotensin–aldosterone system inhibitors (RAASi), which include ACE-inhibitors (ACE-I), angiotensin receptor blockers (ARB) and angiotensin receptor/neprilysin inhibitors (ARNI). Other therapeutics include beta-blockers (BB), mineralocorticoid receptor antagonists (MRAs) and sodium glucose cotransporter-2 inhibitors (SGLT2i) [19]. In HF exacerbation, especially in volume overload, the patients may require diuretics, oxygen therapy, and hospitalization. These patients can be treated with cardiac resynchronization therapy (CRT) or even heart transplantation [22,23]. New treatment strategies include transthyretin stabilizers, intravenous iron for treatment of deficiency, cardiac myosin activators, soluble guanylate cyclase stimulators, and new potassium binders [24]. Despite significant progress in the identification of innovative pharmaceutical treatments for HF over the last few decades, the prevention of premature mortality has only modestly improved [25]. Deteriorating environmental factors, poor diet, and an aging population result in an ever-increasing number of people affected by these diseases. Current treatment methods do not induce cardiac tissue regeneration and are insufficient in reducing fibrotic tissue. For this reason, the development of new treatment strategies and their implementation into daily practice are required as soon as possible. The use of stem cells or heir paracrine factors could become a novel treatment strategy.

## 3. Stem Cells—Differentiation Potential, Tissue Sources and Regenerative Properties

Stem cells represent a unique group of cells that exhibit a remarkable ability of self-renewal and differentiation into diverse cell lineages. They play key roles in neonatal development and constitute the source of specialized cell types in all tissues and organs [26]. In adulthood, stem cells are a crucial element in processes of restoration and regeneration. The activation and recruitment of stem cells is an important phase in regenerative processes after injuries [27]. 

Stem cells can be divided into several groups by their origin and potential for differentiation. Totipotent stem cells have the greatest differentiation potential, as they can form embryonic tissues, as well as extra-embryonic yolk sac and placenta [28]. Pluripotent stem cells can differentiate into cells of all three germ layers and do not form extra-embryonic structures. They are present at various phases of human growth [29]. Multipotent stem cells can form all cell types belonging to the same cell lineage [30]. Oligopotent stem cells differentiate into several cellular subtypes within a specific tissue. Unipotent stem cells have the narrowest differentiation potential. They can repeatedly divide and form only one specific cell type [1]. 

Depending on their development stage, stem cells can be classified as embryonic stem cells and adult stem cells. The latter cells can be found in differentiated tissues and their role is to proliferate and differentiate to renew the specific tissue [31]. Stem cells can be obtained from different sources, which determine their special characteristics and advantages. Mesenchymal stem cells (MSCs) constitute a vast group of cells which are classified into several main groups based on their origin. Bone marrow-derived mesenchymal stem cells (BM-MSCs) have high regenerative and immunomodulatory potential and have been widely tested in clinical trials, which have shown their safety and efficacy [32]. Adipose tissue is a rich source of mesenchymal stem cells (AD-MSCs), which can be easily obtained via subcutaneous lipoaspiration [33]. Mesenchymal fetal stem cells can also be obtained from placenta, umbilical cord (UC-MSCs), as well as amniotic fluid and amniotic membrane [34]. Dental pulp is a source of MSCs with neurotropic properties, as they originate from the neural crest [35]. Periosteum and synovial fluid have been identified as sources of MSCs and their applications could stimulate bone and cartilage regeneration [36,37]. Stem cells are also present in the skin (S-MSCs); these cells stimulate wound healing [38]. Furthermore, it is possible to genetically reprogram somatic cells to achieve stem cell properties. Such induced pluripotent stem cells (iPSCs) can proliferate and differentiate into any cell lineage [39].

Stem cells secrete various extracellular vesicles (EVs): apoptotic bodies, microvesicles and exosomes, which act as paracrine mediators. They may serve as novel therapeutic agents due to their ability to transport various molecules to specific cells, including medications, therapeutic genes, enzymes, and RNA [40]. Stem cell-derived exosomes may contain various inflammatory, fibrotic and angiogenic mediators, such as interleukin-6 (IL-6), interleukin-10 (IL-10), transforming growth factor-β1 (TGF-β1), as well as vascular endothelial growth factor (VEGF) [41]. As the substances they secrete can promote proliferation, inhibit apoptosis, and reduce oxidative stress, they stimulate cell regeneration and inhibit inflammatory processes [42]. Novel cell-free therapies based on EVs may bring major progress in the treatment and management of various autoimmune and inflammatory diseases [43]. In the next paragraphs, we will focus on the potential role of various types of stem cells and their paracrine products in the treatment of CVDs. 

## 4. Stem Cells and Coronary Artery Disease

### 4.1. Stem Cells and Atherosclerosis—Macrophages

Atherosclerosis is a common inflammatory vascular disease characterized by the accumulation of lipoproteins, lipids, fibrous elements, and calcifications within the inner lining of large arteries. Regulating blood lipid levels with the available medications is inadequate due to the increasing prevalence of atherosclerosis and its associations with adverse effects. In the context of vascular atherosclerosis, MSCs exhibit antiapoptotic and anti-inflammatory properties [44]. They have immunomodulatory properties and regulate the behavior of immune cells, including macrophages, which play an important role in atherosclerosis. Macrophages are typically divided into two main subtypes: M1 (classically activated) and M2 (alternatively activated) macrophages [45]. However, recent studies demonstrated that there are more subpopulations of these cells. In atherosclerotic plaques, pro-inflammatory, foamy anti-inflammatory, as well as resident macrophages were detected [46]. 

Studies have demonstrated that MSCs have the ability to regulate the progression of atherosclerosis by mediating macrophage polarization. Fan et al. demonstrated that AD-MSCs transplantation decreased the levels of triglycerides (TG), total cholesterol (TC), and low-density lipoprotein cholesterol (LDL-C) in the serum of rats with atherosclerosis [47]. In line with this observation, similar findings were noted in a study conducted on New Zealand rabbits. Allogeneic AD-MSCs were implanted into rabbits every two weeks for three months following one month of a high-fat diet. The use of stem cells was associated with a significant decrease in LDL-C, TC, and TG levels in the third month compared to the control group. Additionally, ultrasound examinations revealed mitigation of atherosclerotic plaque formation in stem cell-treated animals. In the later stages, reductions in the size of atherosclerotic lesions and inhibition of aortic inflammatory responses were observed in rabbit aorta sections [48]. The influence of MSCs on lipid levels may depend on the stimulation of liver functionality. Intriguingly, an appropriate combination of cytokines can stimulate AD-MSCs to exhibit hepatogenic and angiogenic abilities, which could mitigate the effects of liver fibrosis [49]. In 2018, Takafuji et al. investigated the impact of the condition medium (CM) from cultured MSCs on low-density lipoprotein receptor-knockout (Ldlr^−/−^) mice fed with a high-fat diet. Stem cells significantly inhibited atherosclerotic plaque development by reducing the expression of adhesion molecules and macrophage accumulation in the vessel walls. However, administration of MSC-CM did not significantly affect the change in serum lipoprotein levels. MSC-CM supernatant reduced lipopolysaccharide-induced expression of M1 markers by inhibiting both the mitogen-activated protein kinase (MAPK) and nuclear factor kappa B (NFκB) pathways, as well as increasing the expression of M2 markers through the activation of signal transducer and activator of transcription 3 (STAT3) pathway [50]. The ability of AD-MSCs to promote macrophage switch towards the M2 phenotype was confirmed in another in vitro study [48]. Additionally, AD-MSCs have been found to reduce the secretion of tumor necrosis factor alpha (TNF-α) by the M1 macrophages. These findings imply that AD-MSCs protect against atherosclerosis by targeting M1 macrophage foam cells, through the regulation of the NF-κBp65-TNF-α pathway [48,51].

Human amnion MSCs are easily accessible, pose a low risk of tumor formation, exhibit low immunogenicity, and possess strong paracrine functions. Injection of these cells into the tail vein of male C57BL/6 apolipoprotein E knockout (apoE-KO) mice fed with a high-fat diet alleviated the progression of atherosclerosis by reducing macrophage accumulation and suppressing the inflammatory response in the aortic arteries. Stem cells were found to regulate the secretion of TNFα and interleukin-10 (IL-10) through the NF-κB pathway [52]. Gingival MSCs (GMSCs) are another type of stem cells that mediate macrophage polarization and promote the M2 phenotype, potentially by suppressing indoleamine 2,3-dioxygenase (IDO) and the CD73 signaling [53]. Moreover, S-MSCs exhibit functional similarities to BM-MSCs, but they are significantly easier to access. These skin-derived cells inhibit atherosclerotic plaque development by modulating macrophage function. Specifically, S-MSCs intravenously administered to apoE-KO mice through the tail vein were associated with stimulated macrophages releasing prostaglandin E2, thereby enhancing the release of anti-inflammatory IL-10 while reducing the secretion of TNF-α [54]. Table 1 summarizes the preclinical models and findings regarding the role of MSCs in suppressing atherosclerosis progression by regulating macrophage functionality.

### 4.2. Stem Cells and Atherosclerosis—Endothelial Cells

Apart from macrophages, endothelial cells represent the other highly significant cellular population in the pathogenesis of atherosclerosis. Due to various factors, such as disturbed flow, oxLDL, and pro-inflammatory cytokines, endothelial cells cannot maintain homeostasis, which leads to their dysfunction. Consequently, these cells are activated and the vessel wall stimulates processes including platelet activation, leukocyte adhesion, vasoconstriction, and lipid accumulation [55,56]. 

Multiple studies have demonstrated the efficacy of stem cells in reducing the inflammation in endothelial cells, which translates into relieved atherosclerosis. Specifically, paracrine products, such as extracellular vesicles, play an important role in this process. For example, high glucose conditions impair human umbilical vein endothelial cells (HUVECs) functionality, such as migration and tube disruptions. The condition medium of MSCs could significantly improve these processes [57]. Furthermore, other studies have focused on evaluating the RNA molecules that affect endothelial cell functionality. Stem cells secrete vesicles containing small RNA molecules or can affect the expression of these in endothelial cells. Interestingly, Xiao et al. performed microRNA (miRNA) sequencing of extracellular vesicles derived from MSCs. The authors detected over one thousand miRNA molecules, among which almost four hundred had at least 100 read counts [58]. MiRNAs belong to the family of non-coding RNA (ncRNA), molecules that regulate gene expression. Specifically, miRNAs bind to their target mRNA to suppress its expression. Exosomes derived from AD-MSCs were found to suppress the expression of miR-342-5p, which has been associated with enhanced apoptosis of HUVECs [59]. Furthermore, using extracellular vesicles, MSCs can deliver miR-146a to HUVECs, which reduces senescence of the endothelial cells [58]. Importantly, Li et al. examined the effects of MSCs on endothelial cells stimulated with oxLDL. Stem cells could restore the activity of the Akt/eNOS axis, which was disrupted by the oxLDL. In animal models, the application of MSCs significantly reduced plaque area in the aorta [48,60]. Moreover, MSCs secrete exosomes containing long non-coding RNA (lncRNA) molecules, another members of the ncRNA family. For example, MSCs secrete fetal-lethal non-coding developmental regulatory RNA (FENDRR). These vesicles enhance viability of endothelial cells and promote their pro-angiogenic features. Importantly, these exosomes are associated with significantly reduced plaque area in mice. Mechanistically, FENDRR could bind to miR-28, and thus upregulate TEA domain transcription factor 1 (TEAD1) [61], a molecule implicated in the mitochondrial biogenesis and endothelial angiogenesis [62]. MSCs or their secreted factors could modulate other mechanisms associated with endothelial cells as well. For example, they could suppress platelet activation, which is implicated in the formation of atherosclerotic plaques [63]. 

### 4.3. Myocardial Infarction and Ischemic Heart Disease

#### 4.3.1. Mesenchymal Stem Cells

Ischemic heart disease (IHD) is a clinical syndrome characterized by insufficient supply of oxygen and nutrients to cardiomyocytes. Atherosclerosis of the coronary arteries is considered to be the primary cause of IHD, leading to ischemia through the narrowing of the lumen of the epicardial arteries that supply the heart muscle. IHD has long been the leading cause of death worldwide, and its significance in public health continues to grow. Deaths due to IHD can be broadly categorized into sudden cardiac deaths and deaths resulting from acute myocardial infarction (AMI) [64]. Due to the destruction of cardiomyocytes in many patients after AMI, there is a reduction in LVEF and the associated development of ischemic HF. It is important to emphasize that despite the current broad possibilities in the application of reperfusion therapies such as PCI (percutaneous coronary interventions), reduced LVEF in the acute phase of MI remains the most crucial independent prognostic factor for poor outcomes [65]. Due to the limited regenerative capacity of cardiomyocytes, researchers are closely examining the potential use of stem cells and their regenerative and anti-inflammatory properties in the therapy of myocardial ischemia.

The role of MSCs in the regeneration of the myocardium after MI has been a subject of research and discussion for many years. Potential reparative mechanisms include stimulating angiogenesis, influencing the microenvironment to promote cardiac tissue repair, modulating the immune response, reducing inflammation, and decreasing the scar tissue to potentially enhance the functional recovery of the heart after MI [66]. Preclinical studies have demonstrated important potential benefits of using stem cells in the therapy of ischemia and infarction by modulating various pathways and cellular processes. Paracrine factors secreted by MSCs play a significant role in inducing beneficial effects. For example, an MSC-conditioned medium could affect the functionality of cardiac fibroblasts by increasing the activity of matrix metalloproteinases and suppressing the tissue inhibitor of matric metalloproteinases, thus affecting the extracellular matrix remodeling [67]. 

**Figure 1:**
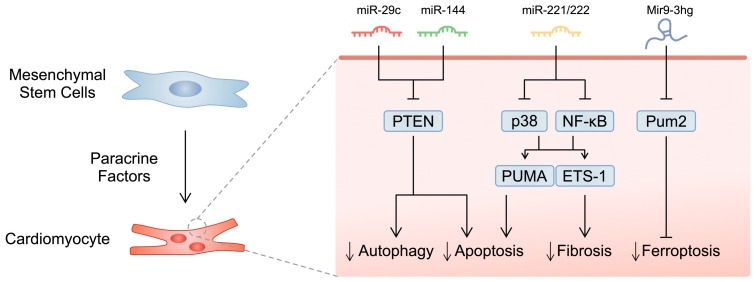
A schematic illustration of selected studies demonstrating the impact of microRNA molecules secreted by mesenchymal stem cells on cardiomyocytes.

MSCs secrete exosomes that promote the survival of cardiomyocytes. Mechanistically, cardiomyocytes accumulate MSC-derived extracellular vesicles containing miR-144, an RNA molecule that targets and inhibits phosphatase and tensin homolog (PTEN), thus suppressing apoptosis [68]. A similar mechanism has been confirmed in another study, where BM-MSC-derived exosomes containing miR-29c could reduced infarct scar formation in vivo. In these experiments, the molecule was also found to target PTEN and suppress excessive autophagy [69]. Furthermore, the condition medium of stem cells from adipose tissue contains miR-221/222, a molecule which could suppress the expression of apoptotic p53-upregulated modulator of apoptosis (PUMA) and fibrotic E26 transcription-specific 1 (ETS-1) through the p38 and NF-κB pathways. Mice treated with a stem cell condition medium demonstrated improved cardiac parameters (EF, fractional shortening), and reduced infarct size [70]. In another study, Zhang and colleagues reported that lncRNA Mir9-3hg is abundantly expressed in exosomes derived from BM-MSCs. It could bind to and inhibit PUM2, thus suppressing ferroptosis of cardiomyocytes [71] (Figure 1). Other molecules detected in MSC-derived exosomes that induce beneficial effects in cardiomyocytes include miR-23a- 3p [72], miR-214 [73], miR-205 [74], and HAD2-AS1 [75]. 

In studies incorporating animal models, transplantation of AD-MSCs improved LVEF in post-MI cardiac failure, in contrast to BM-MSCs or bone marrow mononuclear cells [76,77]. However, in a study by Rasmussen et al. [76] the authors observed that the use of AD-MSCs and BM-MSCs did not increase vessel density, which could result from more advanced age and the burden of coronary artery disease in the donor [78]. 

Epicardial adipose tissue-derived stem cells (EATDS) are phenotypically the most closely related to cardiomyocytes, and due to their low immunogenicity, they hold significant promise for future allogeneic transplantation. Additionally, they exhibit a higher cardiomyogenic potential compared to AD-MSCs [79]. In 2022, Thankam et al. showed that extracellular vesicles secreted by ischemia-stimulated EATDS increase the expression of primary cardiac cells transcription factors (GATA4, Nkx2.5, IRX4, TBX5) in cardiac fibroblasts [80]. The cardiomyogenic potential of EATDS has also been presented in animal studies. Specifically, Özkaynak et al. analyzed the efficacy of EATDS in rabbit MI models. In the therapeutic group, each rabbit received one million (10 × 106 in 100 μL) allogeneic EATDS four weeks after MI induction, administered intramuscularly in the peri-infarct zone. After a further four weeks, this group demonstrated a reduction in the peri-infarct necrotic focus, an increase in vascular density, and a clinically significant improvement in EF [80].

UC-MSCs represent another group of cells with promising regenerative efficacy. These cells develop from an extra-embryonic mesoderm at early stages of embryogenesis and, hypothetically, could have greater differentiation potential. In animal MI models, introduction of UC-MSCs was associated with reduced infarct size, fibrosis regression, cardiac functionality, as well as stimulation of vascular density [81,82,83]. Apart from cardiomyocytes, umbilical cord-derived cells were found to differentiate towards endothelial cells and smooth muscle cells, which could therefore translate into their efficacy in animal infarction models [81]. Despite direct effects of UC-MSCs, their paracrine effects have also shown promising results. Specifically, beneficial effects were observed in animals injected with UC-MSC-derived exosomes [84]. Extracellular vesicles could regulate various mechanisms that ultimately promote cardiac regeneration, such as enhancement of the M2 macrophage polarization [85], as well as modulation of the fibrosis pathways [86]. Inside the umbilical cord, there is a stromal region known as Wharton’s jelly, which is also a source of MSCs (WJ-MSCs). These cells share similarities with embryonic stem cells regarding the expression of stemness markers. In addition, WJ-MSCs express a number of cardiac transcription factors [87], highlighting their potential regenerative role in cardiac disorders. In mini swines, an injection of WJ-MSCs into the ischemic region significantly promoted cardiac functionality (LVEF) and myocardial perfusion [88].

Stem cells have also been investigated in clinical settings (Table 2). Stem cell transplantation can be either autologous or allogeneic. The POSEIDON trial, which evaluated these two cellular treatment methods in patients with ischemic cardiomyopathy, demonstrated that both strategies were safe [89]. In a multicentre clinical trial conducted in 2004–2005, German researchers intravenously administered autologous BM-MSCs to 101 individuals in the study group 3–7 days after successful reperfusion therapy and stent implantation. After four months, it was observed that LVEF was significantly higher in the study group compared to the placebo group. The most significant improvement in the left ventricular contractility size was noted in individuals with the lowest initial LVEF [90]. However, several meta-analyses have demonstrated a broader investigation into the effectiveness of BM-MSCs in post-MI patients [91,92]. Recently, Hosseinpour et al. published a meta-analysis of 10 clinical trials investigating the use of BM-MSCs in patients after AMI. The authors found that MSCs significantly improved LVEF (weighted mean difference WMD = 3.71%, *p* < 0.001). Furthermore, their efficacy was greater compared to bone marrow mononuclear cells. However, the authors did not find significant differences in the other parameters of left ventricular end-diastolic volume (LVEDV) and left ventricle end-systolic volume (LVESV). Nevertheless, these observations became significant in a sensitivity analysis, which only included studies evaluating an introduction of cells within 11 days of AMI [93]. CHART-1 was a prospective, randomized, and one of the largest clinical trials investigating the safety and efficacy of cardiopoietic stem cells. One hundred and twenty patients received the cell-based treatment, and one hundred and fifty-one patients were in the sham cohort. The primary endpoint was an improvement in a composite of several functors, including mortality, HF worsening, and six-minute walk test results. The primary endpoint was not achieved in the overall population. However, better outcomes were observed in a subgroup of patients with an LVEDV of 200–370 mL [94]. Importantly, in the cohort of patients with advanced left ventricular enlargement, a significant improvement in quality of life was observed [95]. Additionally, recent clinical trials did not demonstrate the previously expected improvements after AD-MSCs therapy in patients after MI. Specifically, a phase II clinical trial (SCIENCE) included 133 patients, of which 90 received cell-based therapy. A single intramyocardial injection of AD-MSCs did not significantly improve cardiac functionality, nor mean time to the occurrence of a cardiac adverse event [96]. Similar observations were also made in a recent Danish trial [97]. In another randomized clinical trial, an intracoronary application of WJ-MSCs in patients after MI was examined. Fifty-eight patients received the cellular treatment. Importantly, the allogeneic cellular treatment promoted cardiac functionality and reduced infarct size. Additionally, WJ-MSCs affected the LVEDV and LVESV, therefore preventing cardiac remodeling. Importantly, both cohorts did not differ in terms of major adverse cardiac events [98]. Interestingly, the beneficial properties of WJ-MSCs could be further augmented by introducing a repeated dose, as evidenced by Attar et al. [99]. Specifically, the authors observed that repeating the dose of WJ-MSCs after 10 days was associated with improved cardiac parameters and reduced infarct size. Recently, Prat-Vidal and colleagues [100] described the use of WJ-MSCs supported by decellularized pericardium. Scaffolds represent an important mechanism in tissue engineering that improves cell survival and retention. The authors report the application of WJ-MSC-based tissue graft during coronary artery bypass graft (CABG) procedure, and it was associated with reduced infarct size and changes in ventricular parameters after three months. 

#### 4.3.2. Induced Pluripotent Stem Cells

Induced pluripotent stem cells were introduced for the first time by Yamanaka and collaborators, who reprogrammed mouse cells in 2006 [101]. A year after this discovery, two independent teams demonstrated the first generated iPSC lines. This was achieved by reprogramming fibroblasts into iPSCs using the Oct4/Sox2/Klf4/c-Myc and the Oct4/Sox2/Nanog/LIN28 factors [102,103]. The following studies have shown that it is possible to produce iPSCs from fibroblasts without the involvement of c-Myc [104]. They are somatic-derived cells that are reprogramed to enter the undifferentiated phase by gene transfer using trans-acting factors. Induced stem cells are able to produce cells representing derivatives from three germ layers [102]. Human iPSCs (hiPSCs) have attracted great interest in cardiovascular research due to their ability to differentiate into smooth muscle cells (SMCs), endothelial cells (ECs), and cardiomyocyte lineages [102,105,106,107,108]. Specifically, iPSC-derived cardiomyocytes can be differentiated into atrial and ventricular subtypes [109]. Cardiac differentiation depends on the cellular origin, as iPSCs derived from certain cells reprogram towards cardiomyocytes more easily [110]. Human iPSC-derived cardiomyocytes may serve as a novel therapeutic approach to regenerate cardiac tissues after cardiac impairment. However, the implementation, survival, and engraftment of human iPSCs are challenges that still need to be investigated. 

The administration of iPSCs in animal models of MI has been associated with beneficial outcomes. For instance, Nelson et al. showed that hiPSCs therapy restored myocardial function after acute MI and attenuated remodeling progression [111]. As iPSCs can differentiate towards cardiomyocytes, the use of iPSC-derived cardiomyocytes have been investigated as well. First, iPSC-cardiomyocyte transplantation is associated with significant cardiac regeneration after MI in animal studies [112,113]. Compared to MSCs, iPSC-derived cardiac cells are associated with improved antifibrotic properties [114] and enhanced vasculogenesis [115]. The pro-angiogenic abilities may result from an abundant expression of alpha-B crystallin (CRYAB), which enhances tube formation and migration of endothelial cells [116]. Moreover, in a study by Stępniewski et al., the authors demonstrated that iPSC-derived cardiomyocytes showed greater efficacy in improving cardiac functionality than AD-MSCs in murine MI models [117]. Similarly, beneficial effects were observed after the use of iPSC-cardiomyocyte-derived exosomes, which improved cardiac function and reduced fibrosis by enhancing autophagy [118]. Furthermore, these structures enhance angiogenesis, as stimulation of endothelial cells with exosomes derived from iPSC-cardiomyocytes stimulates the expression of pro-angiogenic markers such as VEGF, platelet-derived growth factor (PDGF), and fibroblast growth factor 2 (FGF2) [119]. Importantly, the use of iPSCs is associated with concerns regarding tumorigenesis. Experiments on rats showed that monitoring the LIN28 marker, which indicates undifferentiated iPSCs, may be crucial to evaluate tumorigenic potential. Specifically, transplantation of iPSC-derived cardiomyocytes with a LIN28-positive fraction greater than 0.33% was associated with tumor formation in rats [120]. One method that could prevent neoplastic tissue development is to eliminate undifferentiated iPSCs. Sougawa and collaborators demonstrated that treatment of iPSC-derived cardiomyocytes with brentuximab vedotin, an agent targeting CD30, could induce apoptosis in residual undifferentiated iPSCs [121]. Irradiation is another method that has been suggested to reduce the risk of teratoma formation [122]. These types of treatments aim to increase the safety of clinical applications of iPSC-derived cardiac cells. However, they may also interfere with cardiomyocyte functionality. Specifically, radiation was found to significantly alter the expression of hundreds of genes in iPSC-derived cardiomyocytes [123]. 

Importantly, iPSC-derived cardiomyocytes demonstrate an immature phenotype, and studies have been examining potential methods to improve the maturation process. These techniques incorporate tissue engineering, scaffolds, or microtissues incorporating various cell types. Firstly, metabolic pathways play key roles in the processes of cardiomyocytes maturation. Specifically, alteration of the culture medium to include low levels of glucose and higher concentrations of fatty acid enhanced contraction and caused structural improvements of differentiated cardiac cells [124]. This culture modification is associated with energy balance, which depends on the quality of mitochondria. Importantly, the maturation of cardiomyocytes may depend on the expression of Sirtuin 3, a mitochondrial deacetylase that regulates the structure and functionality of these organelles [125]. Furthermore, functionality of iPSC-derived cardiomyocytes may be improved by regulating the mitochondrial respiratory chain. Specifically, heat shock protein 90 (Hsp90) mediates the expression of mitochondrial respiratory chain proteins; thus, regulating its expression can alter metabolic output of cardiomyocytes [126]. Apart from mitochondrial functionality, enhancement of the sarcomere performance could improve physiological activity of engineered cardiac tissues. Inducing the expression of α-myosin heavy chain improved contractile function in iPSC-derived cardiac myocytes [127]. 

Various signaling pathways have been suggested to affect cardiomyocyte maturity. Thousands of genes are differently expressed between adult and fetal, as well as in vitro cardiac samples [128]. Modifying the expression of these genes may impact the phenotype and functionality of generated cardiomyocytes. Suppression of mammalian target of rapamycin (mTOR), a member of the phosphoinositide 3-kinase (PI3K) pathway, has been associated with quiescent state, improved contractility, as well as an upregulation of maturity-related genes in iPSC-derived cardiomyocytes [129]. Another pathway involved in the maturation process is MAPK. Concomitant inhibition of the MAPK and PI3K pathways was also associated with improved maturation [128]. In addition, cardiomyocytes depend on ion channels to be able to exert their functions. Therefore, modulating the expression of ion channels may enhance the maturity and reduce the risk of arrhythmia. In a study by Zhou et al., the authors studied the impact of KCNJ2 gene overexpression. KCNJ2 encodes a potassium channel, and its upregulation stimulated electrophysiological and structural maturation [130]. Another potential mechanism that could be applied to improve iPSC-derived cardiomyocyte maturity is a formation of microtissues involving various cellular subtypes. Paracrine products of these cells can increase cardiomyocyte functionality. These microtissues may involve iPSC-derived cardiac cells (endothelial cells, cardiac fibroblasts) [131] and multilineage cells [132]. The presence of vascular cells offers another important benefit; it enhances the proliferation of iPSC-derived cardiomyocytes [133]. Furthermore, stimulation of spheroid aggregation has also been associated with enhanced maturation [134]. 

Recent studies have been investigating the use of iPSC-derived myocardial tissue patches, engineered cardiac tissue, or scaffolds. These methods could enhance cellular engraftment. For example, the use of iPSCs with patches made from decellularized placenta significantly upregulated genes associated with conductivity, structure, maturation, and metabolism. This strategy could significantly improve cardiac function and reduce infarct size in rats after MI [135]. Moreover, several studies examined the use of such tissues in larger animals. Human iPSC patches incorporating three types of cardiac cells together with a fibrin/insulin-like growth factor-1 (IGF-1) patch significantly promoted cellular engraftment and cardiac function in porcine models. Furthermore, this treatment strategy did not induce cardiac arrhythmias [136]. Gao et al. reported that human cardiac muscle patches (hCMPs) generated from human iPSCs improved heart function by stimulatory effect on cell survival and angiogenesis. The hCMP released exosomes promoting cardiomyocyte survival [137]. In a recent study by Miyagawa et al., the authors studied the efficacy of iPSC-derived myocardial patches in porcine infarction models. The authors observed that transplantation of myocardial patches was associated with significant improvements in cardiac functionality even 12 weeks after the procedure. In addition, iPSC-derived tissue enhanced vascular density of the infarct zone. However, the authors did not find a significant difference regarding the level of interstitial fibrosis. Importantly, the treatment did not cause lethal arrhythmias nor tumor formation during study period [138]. Additionally, studies have examined the use of iPSC-derived cardiomyocyte aggregations, known as cardiac spheroids. For example, transplantation of cardiac spheroids in swine models improved EF 8 weeks after the procedure and significantly reduced infarct size. However, it was also associated with the occurrence of tachycardia [139]. Recently, Vo and colleagues performed a meta-analysis to investigate efficacy and safety of iPSC-derived cardiomyocytes in animal models with IHD. The authors included a total of 51 studies, among which 43 included murine models, while large animals were investigated in 8 studies. The authors demonstrated that cellular therapy was associated with a significantly greater LVEF and fractional shortening (FS). However, no significant differences were noted regarding the mortality and presence of ventricular arrhythmias [140].

As previously mentioned, iPSCs can differentiate towards endothelial cells, which has been investigated in the context of MI. Precisely, exosomes obtained from iPSC-derived endothelial cells have been found to suppress cardiomyocyte apoptosis, improve LVEF, reduce infarct size, and enhance cardiomyocyte contraction in the MI models [141]. Combinational transplantation of iPSC-derived endothelial cells and cardiomyocytes in MI animal models was associated with improved cardiac functionality, smaller infarct area, as well as greater presences of blood vessels [142]. 

## 5. Stem Cells and Dilated Cardiomyopathy

Dilated cardiomyopathy is characterized by ventricular dilation or systolic dysfunction when there is no sign of CAD which leads to heart failure or the death of patients [13]. Current therapy strategies do not always provide the desired efficacy. Stem cells have opened remarkable new avenues for tissue regeneration in multiple heart disease. MSCs may play a therapeutic role in DCM due to their differentiation properties. Preclinical observations have indicated their role in the treatment of cardiac fibrosis. 

Cardiac fibrosis, which results from epithelial-to-mesenchymal transition and myofibroblast activity, is involved in the pathogenesis of DCM [143]. Importantly, this process has been associated with the occurrence of a sudden cardiac death [144]. Stem cells have strong antifibrotic properties, which could be utilized in the treatment of DCM. A study by Zhang et al. demonstrated that treatment with UC-MSCs reduced cardiac fibrosis in DCM rat models. Mechanistically, stem cells were found to suppress the expression of TGF-β1, type III collagen, and p-ERK1/2 [145]. Additionally, a study by Mao et al. indicated that injection of HuMSCs promoted FS and LVEF. Levels of B-type natriuretic peptide (BNP) and cTNI were mitigated after administration of HuMSCs. On the other hand, the expression of the angiogenesis-related factors VEGF, IGF-1, and hepatocyte growth factor (HGF) was promoted [146]. Moreover, the same MSC subtype has also been found to suppress the process of endothelial-to-mesenchymal transition [147]. Cardioprotective features in DCM animal studies were also found in the case of BM-MSCs [148] and AD-MSCs [149]. Beneficial effects in DCM have also been observed after the administration of stem cell-derived exosomes. In mouse models with doxorubicin-induced DCM, exosomes could significantly improve cardiac functionality (LVEF and LVFS), and inhibited apoptosis of cardiomyocytes. Intriguingly, these membrane-bound structures were also found to promote the anti-inflammatory macrophage population through the modulation of the JAK2/STAT6 axis [150]. 

These preclinical studies clearly demonstrate the promising efficacy of stem cell or exosome-based therapies. Consequently, studies have been investigating their use clinically. Since several smaller clinical trials have been conducted, meta-analyses have analyzed the role of cellular therapy more broadly. One of such studies published in 2019 included eight clinical trials. According to this analysis, stem cell-based therapy did not improve mortality, but was associated with increased LVEF and reduced LVESV and LVEDCS [151].

## 6. How to Improve the Activity of Stem Cells in Cardiac Diseases?

### 6.1. Impact of Heart Failure on Stem Cell Functionality

Heart failure can develop as a result of previously described diseases which is associated with heart remodeling and impaired functionality [152]. Several studies have evaluated the benefits of stem cells in preclinical animal models and in patients with chronic HF [153,154]. A recent study by Guo et al. investigated the benefits of administering AD-MSCs into the pericardial cavity of rats with HF. The authors observed that this procedure decreased the expression of α-smooth muscle actin (α-SMA) and brain natriuretic peptide (BNP) while it increased that of C-reactive protein (CRP). Furthermore, intrapericardial MSC injection was associated with an upregulation of myocardial VEGF and decreased expression of IL-6 [155].

Interestingly, populations of certain subtypes of stem cells are increased in patients with HF [156]. However, the state of HF may functionally impair circulating stem cells. In a study by Fortini et al. [157], the authors collected and isolated MSCs from the adipose tissue of patients with HF. These cells showed markers of senescence, as the expression of p16 was significantly elevated in cells derived from HF patients. Moreover, the expression was further enhanced in cells from patients with more severe HF. The authors also analyzed expression of members and targets of the Notch signaling. Specifically, Hey 1 and 2 gene expression, as well as Notch1 protein expression were reduced in patients with HF. Furthermore, the state of HF alters the secretome of MSCs. Specifically, stem cells obtained from animals with left ventricle dysfunction produced significantly more pro-inflammatory cytokines, which was associated with reduced regenerative properties [158]. In addition, HF changes the profile of exosomal miRNAs. In a study by Qiao et al., miR-21-5p was the most dysregulated molecule, and it was downregulated in the vesicles derived from HF cardiac stromal cells. These exosomes demonstrated reduced cardioprotective features compared to the vesicles obtained from healthy hearts [159]. These studies demonstrate that HF may alter stem cells functionality, which highlights the potential of improving their efficacy ex vivo. 

### 6.2. Mesenchymal Stem Cells

Stem cell therapy is a promising concept in the treatment of cardiovascular diseases, but the poor survival of engrafted cells may be associated with their reduced efficacy [160]. The activity and viability of stem cells or their differentiation towards cardiac cells can, however, be enhanced by pretreatment with natural and pharmacological agents, as well as genetic transfection to increase the benefits of stem cell therapy. Furthermore, as HF patients demonstrate functionally altered cells, their stimulation or modification of their environment could further enhance their activity. 

The efficacy of stem cells may depend on adipokines, which are immunomodulatory peptides secreted by the adipose tissue. Adiponectin is one of the most extensively studied adipokine with anti-inflammatory properties. It promotes viability and suppresses apoptosis of BM-MSCs stimulated with flow shear stress [161]. Inhibition of apoptosis could be mediated by the AMPK pathway, which is a major downstream signaling molecule of adiponectin [162]. Several studies have presented the benefits of adiponectin in the context of various cardiac disorders. For example, Nakamura et al. evaluated the role of adiponectin in the treatment of HF and demonstrated that adiponectin stimulates AD-MSCs to secrete exosomes. Administration of stem cells to adiponectin-knockout animals did not provide an expected improvement in heart functionality. Conversely, enhancement of adiponectin levels further improved the beneficial effects of AD-MSCs [163]. In another study on MI animal models, adiponectin further enhanced the beneficial effects induced by MSCs, possibly by stimulating AMPK signaling [164]. Transduction of the adiponectin gene into BM-MSCs could attenuate left ventricular dysfunction and reduce fibrosis in the hearts of diabetic rats, possibly through the inhibition of tumor growth factor beta (TGF-β) [165]. 

Another adipokine that regulates the functionality of stem cells is apelin-13. Pretreatment of BM-MSCs with this peptide promoted mitophagy and, consequently, suppressed apoptosis, oxidative stress, and mitochondrial dysfunction, which could be dependent on the activation of AMPK signaling [166]. Moreover, it stimulates MSCs functionality under hypoxic conditions [167]. In an MI animal model, transplantation of MSCs pretreated with apelin-13 significantly improved EF and left ventricle fraction shortening, and stimulated cells were associated with longer survival at ischemic hearts [168]. Interestingly, myocardial injection of apelin could activate residual cardiac stem cells to decrease infarct size and improve cardiac function [169]. 

Some studies have examined the role of C1q and tumor necrosis factor-related protein 9 (CTRP9), an adipokine and a cardiokine [170], on the functionality of stem cells. Li et al. demonstrated that the addition of CTRP9 into the culture of aged MSCs improved their proliferation and immunoregulatory properties through the AMPK signaling [171]. In cardiac tissue, expression of CTRP9 might determine the beneficial outcomes of MSCs, as delivery of AD-MSCs into CTRP9-knockdown mice was associated with reduced engraftment of stem cells into infarct areas [172]. Furthermore, Liu and colleagues recently demonstrated that CTRP9-281, a C-terminal polypeptide, could promote the secretion of exosomes containing VEGF, thus enhancing angiogenesis [173]. MiR-34a-5p was identified as an upstream inhibitor of CTRP9, and downregulation of this miRNA also enhanced the cardioprotective features of AD-MSCs [174]. Additionally, transplantation of stem cells into the region of infarction is a common method of stem cell delivery in studies investigating their efficacy, but intravenous injection has also been examined. The introduction of stem cells via intravenous delivery has, however, been associated with unfavorable tissue redistribution. Resistin, another adipokine, may be the solution for this obstacle, as it has been found to promote myocardial homing of AD-MSCs, which could explain its stimulation of AD-MSC-mediated cardioprotection [175]. Beneficial effects have also been observed after pretreatment of MSCs with asprosin. This adipokine has been found to stimulate the ERK1/2 pathway to enhance the expression of antioxidant molecules and to inhibit apoptosis, which could translate into improved outcomes of pretreated MSC on the animal model of MI [176]. 

Another molecule suggested to regulate the activity of stem cells is sirtuin 1 (SIRT1), an NAD+-dependent deacetylase that regulates the activity of multiple proteins and thus is involved in metabolic and inflammatory processes [177]. In embryonic stem cells, suppression of SIRT1 activity and siRNA-mediated silencing promotes cell death, as it regulates DNA repair proteins [178]. Apart from its role in cellular survival, SIRT1 takes part in the differentiation of stem cells [179]. The use of SIRT1-knockdown stem cells has been associated with reduced efficacy in the treatment of HF: compared to wild type cells, hearts treated with modified stem cells demonstrated reduced EF. Furthermore, heart tissues from the study group demonstrated lower capillary density and enhanced fibrosis [180]. In addition, pathological conditions may alter SIRT1 expression; for instance; high glucose reduces SIRT1 expression in BM-MSCs [181]. Agents that promote SIRT1 activity have been found to improve the treatment of cardiac conditions. For instance, Chen and collaborators analyzed heart tissues from rats with diabetes and found reduced protein expression of survival markers in this cohort. Transplantation of AD-MSCs could increase their expression, but the use of cells pretreated with resveratrol, a SIRT1 activator, significantly promoted the expression of survival proteins [182]. In another study, resveratrol was found to induce SIRT1 expression in BM-MSCs by suppressing miR-34a. Transplantation of the pretreated cells into the areas of infarction enhanced the expression of the angiogenesis markers: VEGF and HIF-1α [181]. Similarly, pretreatment of aged MSCs with SRTT1720, another SIRT1 activator, significantly improved cardiac function after infarction in animal models. In addition, it suppressed the apoptosis of MSCs through the upregulation of antiapoptotic Fas apoptosis inhibitory molecule (FAIM) [183]. Stimulation of AD-MSCs with melatonin, which also promotes SIRTT1, has been found to reduce apoptosis, improve functionality, and suppress cardiac fibrosis in animal models of myocardial infarction [184].

Moreover, as activation of SIRT1 has been associated with suppression of the progression of HF [185], stem cells could be modified to promote the deacetylase in heart tissue. As previously mentioned, stem cells secrete membrane-bound exosomes, which transport bioactive cargo, such as ncRNA. Transfection of stem cells with plasmids may lead to overexpression of particular transcripts in exosomes. For example, exosomes derived from MSCs transfected with the lncRNA KLF3 antisense RNA 1 (KLF3-AS1) could reduce the infarct zone in rats after myocardial infarction. Mechanistically, KLF3-AS1 binds to miR-138-5p, which ultimately upregulates SIRT1 [186]. Therefore, these studies demonstrate an important role of SIRT1 in the treatment of cardiac diseases. Promotion of SIRT1 enhances survival of stem cells which, in turn, exert more beneficial effects in injured cardiac tissues. 

Several studies have demonstrated the beneficial role of insulin growth factor 1 (IGF-1) on stem cell functionality in various disease models [187,188]. Conversely, blockade of IGF-1R suppresses viability and promotes apoptosis of stem cells [189]. Treatment of BM-MSCs with IGF-1 stimulates their differentiation into cardiomyocyte-like cells [190]. Compared to stem cells alone and the control group, IGF-1 overexpressing adipose-derived stem cells significantly increased EF in rats after myocardial infarction. Secretion of IGF-1 enhanced the PI3K pathway [191], the activation of which has been associated with cardiomyocyte proliferation, as well as regulation of apoptosis and autophagy [192,193]. Importantly, stimulation of MSCs with agents that promote autophagy have demonstrated improved cardioprotective properties [194,195]. For example, stimulation of MSCs with rapamycin upregulated the autophagy marker LC-3 and improved their viability. Pretreated cells enhanced the cardiac function of rats after infarction, as demonstrated by echocardiography. Furthermore, these cells could increase the thickness of the left ventricle and reduce the post-infarct scar. Additionally, rapamycin enhanced the survival of transplanted cells, which also significantly promoted the process of angiogenesis in the area of infarction [194]. 

Peroxisome proliferator-activated receptor (PPAR) β/δ belongs to the family of nuclear receptors that mediate metabolic and inflammatory processes [196]. The activity of PPARβ/δ has been suggested to mediate the immunomodulatory properties of stem cells [197]. In a model of MI, knockdown of PPARβ/δ or the use of its antagonist suppressed the ability of MSCs to reduce infarct size, suggesting that PPARβ/δ has a cardioprotective role. However, it did not alter their secretion of anti-inflammatory cytokines [198]. Conversely, pretreatment of MSCs with PPARβ/δ is associated with greater protection of cardiac cells from oxidative stress and had better efficacy than unstimulated MSCs in an ex vivo model of cardiac ischemia-reperfusion injury [199]. 

Beneficial effects have been observed when MSCs were modified to express integrins, receptors that regulate adhesion. Specifically, these studies investigated the upregulation of integrin-linked kinase (ILK), which interacts with integrins and regulates adhesion. Under hypoxic conditions, overexpression of ILK significantly enhanced MSCs survival and promoted the activity of the Akt and ERK1/2 pathways. Modified MSCs also demonstrated improved adhesion capabilities, and their transplantation into an animal model of MI was associated with reduced fibrosis and infarct size, as well as reduced number of apoptotic cells [200]. ILK may also modify paracrine features of MSCs, as stimulation of cardiac fibroblasts with the condition medium from ILK-MSCs was associated with altered expression of fibrosis-related genes. Moreover, application of this condition medium into MI animals significantly improved cardiac function compared to the medium derived from unmodified MSCs [201]. Other studies have also demonstrated beneficial effects of ILK-MSCs in animal models of MI [202,203]. 

Modulation of the expression of cardiac-specific transcription factors is another strategy that could boost the efficacy of stem cell therapy. As previously mentioned, Nkx2.5 is a marker of cardiac progenitors, and Nkx2.5-positive cells may differentiate towards cardiomyocytes or vascular cells. Various molecules have been shown to regulate the activity of Nkx2.5 and that there are several synergistic factors, such as GATA4 [204]. Regulating the expression of Nkx2.5 has been suggested as a promising therapy of MI [205]. Transfection of BM-MSCs with Nkx2.5 or GATA and their co-culture with cardiomyocytes significantly enhanced differentiation towards cardiac cells. Transplantation of transfected cells has been shown to potentially enhance cardiac repair in a rabbit [206] and mouse models [207]. In addition, Nkx2.5-transfected MSCs could be used in the treatment of HF. In a study by Deng et al., the authors demonstrated that transfected cells were associated with upregulated cardiac markers, while introduction of modified cells into the rat HF models significantly improved EF and fractional shortening, as well as reduced myocardial fibrosis [208]. 

CVDs are a group of diseases in which inflammation plays a role in the pathogenesis. Specifically, atherosclerosis and MI are associated with immune cell responses, including that of T cells [209,210]. Modulation of T cell activity occurs through the programmed death cell 1 pathway (PD-1/PD-L1), which is used in immunotherapy for the treatment of malignancies. In patients with MI, the expression of PD-1 in peripheral blood mononuclear cells (PBMCs) fluctuates in a time-dependent manner, and significantly reduced expression is observed in patients with a more severe infarction. Furthermore, a different expression profile of PD-1 is observed in an animal cardiac tissue in infarcted and its adjacent regions [211]. Injection of AD-MSCs overexpressing Akt and PD-L1 into the infarcted hearts of rats has been shown to promote the end-systolic pressure–volume relationship and preload-recruitable stroke work, which are significantly reduced in animals with MI. The modified stem cells reduced infarct size and promoted CD25+ cells, which play a cardioprotective role during MI [212,213]. In addition, overexpression of PD-L1 has been suggested to improve the viability and survival of stem cell-derived cardiomyocytes by reducing their immunogenicity [214]. 

**Figure 2:**
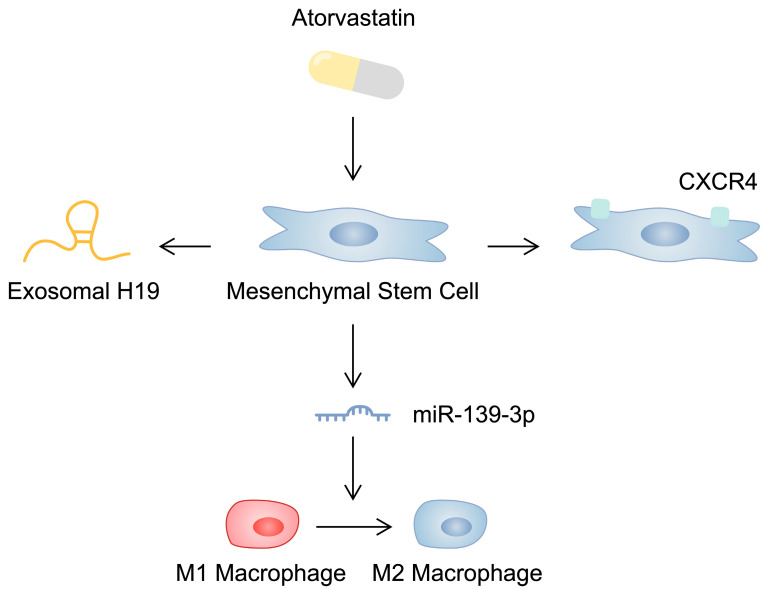
Schematic illustration demonstrating the impact of atorvastatin on mesenchymal stem cells, which could affect their cardioprotective capabilities.

**Figure 3:**
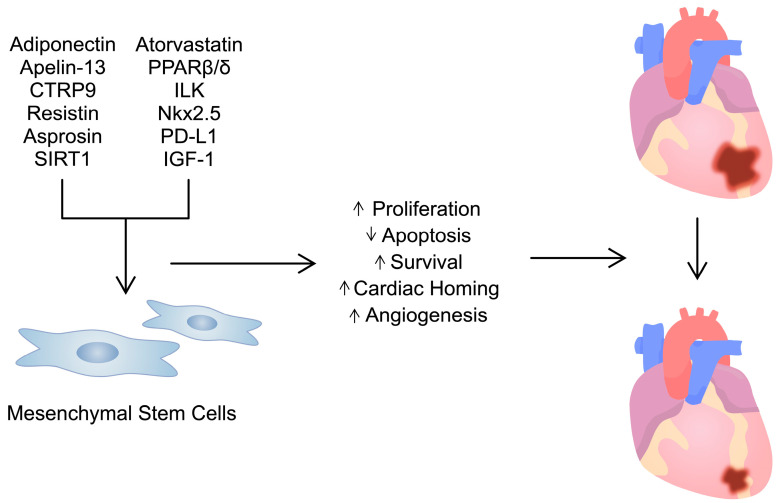
Pretreatment of mesenchymal stem cells with numerous agents/molecules enhances their activity. This could be used in the treatment of cardiac disorders, such as myocardial infarction.

The impact of pretreatment of MSCs with classic pharmacological agents has been investigated. This includes statins, which are widely used to reduce cholesterol concentrations. Stimulation of stem cells with atorvastatin has been found to promote pro-angiogenic mechanisms [215,216]. In addition, pretreatment has been associated with an upregulation of the CXC chemokine receptor 4 (CXCR4) and improved stem cell cardiac homing in animal MI models, as well as increased efficacy, reduced inflammation and fibrosis [217]. Atorvastatin can modulate the secretome of MSCs by changing the cargo of exosomes. Specifically, as demonstrated by Huang and collaborators, atorvastatin enhances the secretion of lncRNA H19 in stem cell-derived exosomes, which could be responsible for the beneficial activity of pretreated stem cells [218]. Several studies have demonstrated the beneficial impact of H19 on cardiac functionality in various models of cardiac disorders [219,220,221,222]. In another report, stimulation with atorvastatin significantly downregulated six miRNA molecules and upregulated three miRNAs in exosomes. An upregulated miR-139-3p could stimulate macrophage polarization towards the M2 anti-inflammatory phenotype, thus improving their effects in an MI model [223] (Figure 2). These promising preclinical study results paved the way for a clinical trial; Yang et al. compared the efficacy of intense atorvastatin therapy combined with mononuclear cell transplantation. The combination cohort achieved a significantly higher LVEF value compared to the group receiving intense atorvastatin, suggesting an important synergy between these methods [224]. On the contrary, statins may also negatively affect the functionality of iPSC-derived cardiomyocytes by reducing their viability and metabolic activity [225]. Pretreatment of MSCs with various other agents has been associated with improved efficacy [226,227]. Overall, pretreatment or transfection of stem cells with numerous respective agents and genes has been associated with improved efficacy in the treatment of CVDs (Figure 3; Table 3). 

We have previously mentioned that stimulation of the M2 anti-inflammatory macrophages could represent a beneficial mechanism in the treatment of atherosclerosis and CAD. Studies have demonstrated that macrophage polarization can be mediated by stem cells. However, Patel et al. report a clinical trial investigating the use of ixmyelocel-T, a product which contains bone marrow cells, including MSCs and increased number of the M2 macrophages. Patients with ischemic DCM underwent a transendocardial injection of the product. Importantly, the treatment composed of cellular combination was associated with a 37% reduction in cardiac events. Furthermore, the cohort administered with placebo significantly more often experienced an AE. However, the cellular treatment did not significantly improve LVESV, LVEDV, LVEF, as well as NYHA class nor 6 min walking test results [228]. 

## 7. Conclusions and Future Perspectives

To conclude, cellular and exosome therapies represent exciting treatment strategies for patients following MI, with cardiomyopathy, or HF, to induce cardiac tissue regeneration and improve organ functionality. Multiple studies have demonstrated beneficial outcomes of treatment incorporating MSCs and iPSC-derived cardiomyocytes, as well as stem cell-derived extracellular vesicles in CVDs. Some of these cells have been examined clinically, but clinical trials have shown a more modest efficacy of these therapies. Several factors could be considered to improve the outcomes of clinical trials, such as including a homogenous study group, administering MSCs with different bioactivity, and delivery method [229]. Furthermore, the effectiveness of stem cells can be enhanced by improving their survival, engraftment, cardiac homing, and pro-angiogenic capabilities, as well as stimulation of cardiomyocyte differentiation. These processes could be achieved by regulating circulating adipokines, pretreatment of stem cells with natural or pharmacological agents, as well as gene transfection. These modifications may significantly change the expression profile and secretome of MSCs, thus affecting their immunomodulatory and regenerative properties. 

Currently, iPSCs are considered as highly attractive in the field of cardiac regeneration. Several clinical trials are being conducted that aim to investigate the safety and efficacy of iPSC-derived cardiomyocytes (e.g., NCT05566600 [230], NCT04396899 [231], NCT04945018 [232]). Numerous studies investigated methods to improve the maturation of iPSC-derived cardiomyocytes, which may eventually lead to the improved efficacy of cardiac tissues formed by these cells. Moreover, treatment with iPSC-derived cardiomyocytes can also be enhanced with the use of natural agents [233]. Importantly, engineered heart tissues incorporating iPSC-derived cardiomyocyte provide opportunities to study drug testing and disease mechanisms, which may also translate into improved treatment outcomes [234]. Recently, Yang and colleagues generated chambered and vascularized cardiac organoids, which is highly promising for the above-mentioned purposes [235]. Interestingly, Cai et al. developed a protocol to differentiate iPSCs towards heart valve cells, creating further opportunities to study valvular heart diseases [236]. Furthermore, iPSC-derived cardiomyocytes from individuals carrying genetic variants associated with the occurrence of cardiomyopathy enable studying the importance of these mutations in disease pathogenesis [237]. In addition, iPSC-derived cardiac cells can serve as important models to study the cardiotoxicity of drugs, such as doxorubicin. For example, iPSC-derived cardiomyocytes may be used to study cellular responses to the cardiotoxic drug [238]. Moreover, as demonstrated by Magdy and collaborators, iPSC-derived cardiomyocytes can undergo gene editing to study the impact of particular variants on doxorubicin-induced cardiotoxicity [239]. Future studies are required to investigate the beneficial mechanisms induced by stem cells and how to improve their cardioprotection and regenerative properties. 

## Figures and Tables

**Table 1:** Summary of selected studies demonstrating beneficial role of various stem cells in animal models of atherosclerosis by regulating the functionality of macrophages.

Stem Cells	Animal Model, Stem Cell Introduction	Impact of Stem Cells on Macrophage Function in the Context of Atherosclerosis	References
MSC-CM	Ldlr^−/−^ mice, intravenous injection	MSC-CM could reduce the formation of atherosclerotic plaque by suppressing macrophage accumulation in the blood vessel wall.	[50]
AD-MSCs	New Zeleand rabbits, intravenous injection	Administration of AD-MSCs was associated with suppressed plaque formation and reduced accumulation of the M1 macrophages and pro-inflammatory mediators.	[48]
Amnion MSCs	C57BL/6 apoE-KO mice, intravenous injection	Stem cells suppressed the progression of atheroclerosis by inhibiting the accumulation of macrophages.	[52]
GMSCs	ApoE^−/−^ mice, intravenous injection	GMSCs suppress the progression of atherosclerosis and inhibit the formation of foam cells; furthermore; they enhance the M2 anti-inflammatory macrophages.	[53]
S-MSCs	ApoE^−/−^ mice, intravenous injection	Stem cells promoted the anti-inflammatory responses of macrophages.	[54]

MSC—mesenchymal stem cell; AD-MSC—adipose-derived mesenchymal stem cell; GMSC—gingival-derived mesenchymal stem cell; S-MSC—skin-derived mesenchymal stem cell; CM—condition medium; apoE—apolipoprotein E; Ldlr—low-density lipoprotein receptor.

**Table 2:** Summary of selected clinical trials investigating the role of stem cells in myocardial infarction or ischemic heart disease.

Stem Cells	Number of Patients	Efficacy of Cellular Treatment	Selected Adverse Events	References
Cardiopoietic cells (conditioned MSCSs)	Cardiopoietic cells = 120	The primary endpoint was neutral for the whole population Significant improvement in patients with an LVEDV of 200–370 mL	Cardiopoietic cells Any AE: 20.8% Any serious AE: 14.1% Death: 8.3%	[94]
	Control = 151		Control Any AE: 5.3% Any serious AE: 1.2% Death: 8.2%	
WJ-MSCs	WJ-MSCs = 58	Cellular treatment promoted LVEF increase, together with LVESV and LVEDV decrease	WJ-MSCs: Rehospitalization for heart failure: 1.7% Ectopic tissue formation: 1.7%	[98]
	Placebo = 58		Placebo Rehospitalization for heart failure: 0% Ectopic tissue formation: 1.7%	
WJ-MSCs	70 (single intervention = 20; repeated intervention = 20; control group = 25)	LVEF increased, LVESD decreased	No adverse events were reported.	[99]
AD-MSCs	AD-MSCs = 90;	LVESV, LVEDV and LVEF did not change	AD-MSCs Heart failure worsening: 15.5% Ventricular tachycardia/fibrillation: 6.6% Myocardial infarction: 4.4% PCI or CAGB: 2.2% Stroke or TIA: 1.1% Angina worsering: 1.1% Death: 3.3%	[96]
	Placebo = 43		Placebo Heart failure worsening: 16.3% Ventricular tachycardia/fibrillation: 0% Myocardial infarction: 2.3% PCI or CAGB: 0% Stroke or TIA: 2.3% Angina worsering: 2.3% Death: 4.7%	
AD-MSCs	AD-MSCs = 54	LVEF, LVESV and LVEDV	AD-MSCs Heart failure worsering: 9.3% Ventricular fibrillation/tachycardia: 1.9% Myocardial infarction: 3.7% Atrial fibrillation: 3.7% Angina worsening: 9.3% Cancer: 1.9% Death: 5.6%	[97]
	Placebo = 27		Placebo Heart failure worsering: 7.4% Ventricular fibrillation/tachycardia: 3.7% Myocardial infarction: 3.7% Atrial fibrillation: 3.7% Angina worsening: 3.7% Cancer: 0% Death: 0%	

AE—adverse event; AD-MSC—adipose-derived mesenchymal stem cell; MACE—major cardiac adverse event; WJ-MSCs—Wharton Jelly-derived mesenchymal stem cell; LVESV—left ventricular end-systolic volume; LVEDV—left ventricular end-diastolic volume.

**Table 3:** Summary of methods to improve the functionality of mesenchymal stem cells.

Agent/Molecule	Mechanisms Mediating the Functionality of Stem Cells	References
Adiponectin	Stimulation of circulating adiponectin promotes the beneficial effects of MSCs in HF.	[163]
	Adiponectin further enhanced the beneficial effects of MSCs in the treatment of animal models with cardiac infarction.	[164]
	Adiponectin transduction into BM-MSCs could enhance the positive effects on left ventricle and fibrosis in diabetic rats.	[165]
Apelin-13	MSCs pretreated with apelin-13 were associated with improved viability and could further increase cardiac repair after infarction in animal models.	[168]
CTRP9	Injection of AD-MSCs into CTRP9-knockdown mice with myocardial infarction was associated with reduced engraftment.	[172]
	CTRP9-281, a C-terminal polypeptide, stimulates stem cells to produce exosomes with a pro-angiogenic cargo and further enhances cardioprotection.	[173]
	Inhibition of miR-34a-5p, an upstream inhibitor of CTRP9, could further enhance the cardioprotective role of adipose-derived stem cells.	[174]
Resistin	Resistin promotes homing of MSCs towards cardiac tissue and thereby improves their cardioprotective potential.	[175]
Asprosin	Pretreatment of MSCs with asprosin stimulated the ERK1/2 pathway to upregulate antioxidant molecules and suppress apoptosis, which could translate into elevated cardioprotection of pretreated stem cells.	[176]
SIRT1	SIRT1-knockdown cells demonstrate reduced efficacy in the treatment of HF.	[180]
	Pretreatment of stem cells with resveratrol enhanced the expression of survival proteins in the hearts of rats with diabetes.	[182]
	Pretreatment of stem cells with resveratrol promoted the expression of proangiogenic mediators in hearts after infarction.	[181]
	Aged MSCs pretreated with SRT1720, a SIRT1 activator, had significantly enhanced cardiac function after infarction in animal models.	[183]
	Stem cells with melatonin enhances the expression of SIRT1 and stimulates animal hearts recovery after infarction.	[184]
IGF-1/IGF-1R	Stimulation of BM-MSCs with IGF-1 enhances their differentiation into cardiomyocyte-like cells	[190]
Rapamycin	Pretreatment of MSCs with rapamycin enhanced the cardioprotective properties of these cells, improved their survival, and enhanced angiogenesis at the area of infarct in the rats model of myocardial infarction.	[194]
PPARβ/δ	PPARβ/δ knockdown or the use of its antagonist suppressed the ability of MSCs to reduce infarct size.	[198]
	The use of PPARβ/δ agonist enhances the cardioprotective role of MSCs.	[199]
Integrin-linked kinase	MSCs overexpressing integrin-linked kinase demonstrated greater viability. Transplantation of these cells into an animal model of myocardial infarction was associated with reduced fibrosis and number of apoptotic cells.	[200]
	Integrin-linked kinase modifies the paracrine properties of MSCs, as the condition medium of modified cells could significantly improve cardiac function.	[201]
Atorvastatin	Pretreatment of MSCs with atorvastatin upregulated CXCR4 and resulted in improved cardiac homing of stem cells. Furthermore, the use of pretreated cells could significantly improve cardiac function and lower fibrosis and inflammation.	[217]
	Atorvastatin enhanced the secretion of lncRNA H19 in stem cell-derived exosomes, which was associated with improved cardiac function.	[218]
	Atorvastatin significantly changed the profile of miRNAs secreted by MSCs in exosomes, which could promote the M2 macrophage polarization.	[223]

HF—heart failure; MSC—mesenchymal stem cells; CTRP9—C1q and tumor necrosis factor-related protein 9; IGF-1—insulin growth factor 1.

## Data Availability

Not applicable.
